# Treatment of Cesarean Scar Ectopic Pregnancy in China with Uterine Artery Embolization—A Systematic Review and Meta-Analysis

**DOI:** 10.3390/jcm11247393

**Published:** 2022-12-13

**Authors:** Greg J. Marchand, Ahmed Taher Masoud, Catherine Coriell, Hollie Ulibarri, Julia Parise, Amanda Arroyo, Sydnee Goetz, Carmen Moir, Atley Moberly, Malini Govindan

**Affiliations:** 1 Marchand Institute for Minimally Invasive Surgery, Mesa, AZ 85209, USA; 2 Faculty of Medicine, Fayoum University, Fayoum 63514, Egypt

**Keywords:** cesarean scar, uterine artery embolization, ectopic pregnancy, extrauterine pregnancy

## Abstract

Cesarean scar ectopic pregnancy (CSP) is a rare form of ectopic pregnancy, and treatment of CSP with uterine artery embolization (UAE) is a novel approach. With increasing numbers of cesarean sections being performed annually, the incidence of this condition is likely to increase. The authors became aware of an unusually high number of published studies originating in mainland China regarding this unusual treatment and sought to perform a meta-analysis to provide comprehensive evidence on this novel practice. Methods: We performed a thorough search and included all forms of quality studies on this topic that reported UAE as a part of first-line management of CSP. We included only studies originating in China. Ultimately, 37 studies were included for qualitative and quantitative synthesis of evidence. After screening retrieved records and extracting data from eligible studies, we pooled continuous data as a mean estimate and 95% confidence interval (CI), and dichotomous data as proportion and 95% CI. Results: CSP patients treated with protocols including UAE had a mean time of 30 days for serum β-hCG normalization, 95% CI [26.816, 33.881]. They had a mean estimated intraprocedural blood loss of 4.19 ± 3.76 mL, a mean hospital stay of nine days, 95%CI [7.914, 9.876], and a success rate of 93.4%, 95%CI [0.918, 0.951]. The severe complication rate was 1.2%, 95%CI [0.008, 0.017]. Conclusion: UAE, in combination with other procedures is being used effectively for the treatment of CSP in China. Protocols including UAE have a success rate of approximately 93.4%, and a severe complication rate of approximately 1.2%. This data’s utility is limited by vast differences in the studied protocols and questionable feasibility outside of China.

## 1. Introduction

Ectopic pregnancy describes pregnancies outside of normal positioning in the uterus, most frequently in the fallopian tube and less frequently in other sites such as the ovaries, abdomen, cesarean scar, and other sites [1]. The incidence of all ectopic pregnancies has increased in recent decades and complicates approximately 2% of all pregnancies, following the increase in the cesarean section rate [1,2].

Cesarean scar pregnancy (CSP) is an ectopic pregnancy located at a previous uterine scar [3]. Its incidence is increasing due to the increased frequency of cesarean sections worldwide [4,5]. It occurs in 1 in 500 pregnancies among women with a previous cesarean delivery and compromises 4% of all ectopic pregnancies [6]. Despite its rarity, CSP can constitute a life-threatening condition [7].

Originally, hysterectomy was considered the only treatment option for CSP [3], however, in recent years, more conservative approaches have been developed. Treatment options now include systemic methotrexate (MTX), uterine artery embolization (UAE), local resection of the ectopic gestational mass, hysteroscopy, and uterine dilation and curettage (D&C) [8,9,10].

Largely used in the treatment of uterine fibroids, uterine artery embolization (UAE) is a widely used procedure generally performed by interventional radiologists under local anesthesia and carried out by catheterization of the uterine arteries through a transfemoral approach. The procedure involves injecting gelatin sponge particles to block the supplying arteries to the uterus, resulting in the cessation of blood supply to the CSP [11]. It may be combined with a dose of MTX given in the intraprocedural period [11,12]. Other authors have reported using polyvinyl alcohol instead of gelatin sponge particles with similar results [13].

UAE may be used alone or combined with local or systemic MTX for treatment of CSP. Moreover, it can be performed before uterine D&C, laparoscopy, hysteroscopy, or local resection [13,14,15,16].

The authors of this study noticed a tremendous increase in published trials that included the use of UAE as a treatment coming out of China. The authors hypothesize that this may likely be due to a decreased regulatory effect on medical care in this country, versus the majority of the rest of the world. As a result, a large body of research on the usage of UAE in CSP from China has surfaced over the last ten years. We aimed to present a global report on the usage of protocols including UAE for the treatment of CSP in China, by conducting this systematic review and meta-analysis.

## 2. Materials and Methods

We conducted this systematic review and meta-analysis guided by the *Cochrane Handbook for Systematic Reviews of Interventions* [17], then we reported it using the “preferred reporting items for systematic review and meta-analysis” (PRISMA statement) [18].

### 2.1. Literature Search

We searched PubMed, Scopus, Web of Science, ClinicalTrials.Gov, MEDLINE, and the Cochrane Central Register of Controlled Trials (CENTRAL) for published studies from inception till April 2021 using the following keywords: “cesarean scar pregnancy,” “ectopic pregnancy”, “extrauterine pregnancy”, “cesarean scar”, “cesarean cicatrization”, and “uterine artery embolization”.

### 2.2. Eligibility Criteria and Study Selection

We included case series, observational studies, comparative studies, and randomized controlled trials (RCTs) that reported UAE as a part of first-line management of CSP, originating from anywhere within China. Exclusion criteria included: (1) Case reports or review articles, (2) case series describing less than five cases managed by UAE, (3) studies where treatment modality or outcomes were not sufficiently detailed, (4) non-English language studies (5), and in vitro or animal studies. After removing duplicates by Endnote, title, and abstract screening, the full-text screening ensured the studies’ eligibility for inclusion. Moreover, we screened the bibliographies of the included studies manually for other relevant studies. Screening was performed independently by two separate authors, and agreement was reached by consensus between the authors. Per our institute standards, a third researcher was assigned to resolve any disputes but was ultimately never needed. Only three studies that met our criteria were excluded because they were located outside of China.

### 2.3. Data Extraction

Extracted data included the year of publication, study design, inclusion period, the mean age of participants, gestational age, primary treatment modality, number of cases per group, success rate, causes of treatment failure, rate of severe complications, time for serum β-hCG normalization, length of hospital stay, intraprocedural blood loss, number of cases undergoing hysterectomy or laparotomy, cases with bleeding more than 500 mL or received a blood transfusion, conclusion, and study country of origin. The management was considered successful if there was no major complication and the patient needed no additional treatments. Severe complications included a UAE procedure that required hysterectomy, laparotomy, involved bleeding >500 mL, or necessitated an unexpected blood transfusion.

### 2.4. Quality Assessment

We used the national institute of health (NIH) tools to assess the quality of cohort and case series studies [19]. For RCTs, we used the Cochrane risk of bias tool described in the Cochrane Handbook for Systematic Reviews of Interventions [17].

### 2.5. Data Synthesis

Analysis was conducted using Open Meta-Analyst software. We reported dichotomous outcomes as a proportion and a 95% confidence interval (CI) and continuous outcomes as a mean estimate and a 95% CI. When heterogeneity was significant (Chi-square *p* < 0.1), we employed the random-effects model and then made a sensitivity analysis to solve the heterogeneity.

## 3. Results

### 3.1. Literature Search Results

The literature search retrieved 433 records; of them, 109 duplicates were removed. We excluded 232 studies during the title and abstract screening and 57 during full-text screening. In addition to the remaining 35 studies, 2 studies were included through the manual search, and a total of 37 studies were included for qualitative and quantitative synthesis of evidence [11,13,14,15,16,20,21,22,23,24,25,26,27,28,29,30,31,32,33,34,35,36,37,38,39,40,41,42,43,44,45,46,47,48,49,50,51,52,53,54] (Supplemental Figure S1). Interestingly, only three studies that would have otherwise met our screening criteria were excluded because they originated in countries other than China.

### 3.2. Characteristic of Included Studies

Included studies are variable in their design, including cohort studies, case series studies, and RCTs with a total of 2655 patients. The most frequent treatment modality in the included studies was UAE combined with D&C or UAE combined with MTX and D&C. Table 1 shows the summary of included studies and the characters of the included patients.

### 3.3. Quality Assessment

The quality of most included studies was fair. The final judgments of each study quality are shown in Table 1, and the details of each quality assessment domain are shown in Supplementary Tables S1 and S2.

### 3.4. Analysis of the Outcomes

#### 3.4.1. Time for Serum β-Human Chorionic Gonadotropin (β-hCG) Normalization (Figure 1)

The time for serum β-hCG normalization (defined as reaching a level of less than or equal to 5 mIU/mL,) was reported by 20 studies in 23 different study groups. The overall mean time for β-hCG resolution to normal level was 29.817 days; 95% CI [26.158, 33.476], and the analysis was heterogeneous (*p* < 0.001, I^2^ = 99%).

**Figure 1:**
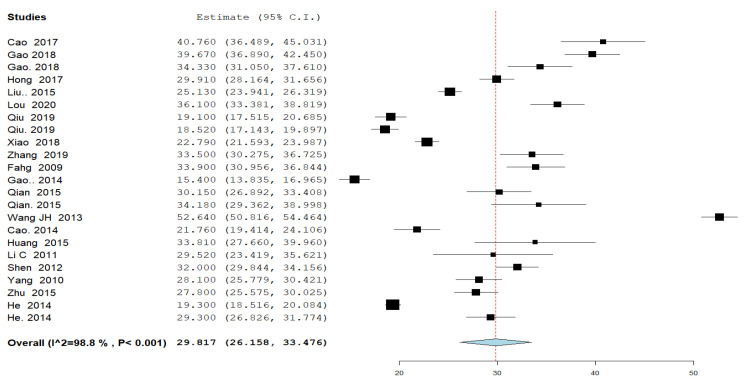
A forest plot of the time for serum β-human chorionic gonadotropin (β-hCG) normalization (defined as reaching a level of less than or equal to 5 mIU/mL,) in days.

#### 3.4.2. Hospital Stay (Figure 2)

Thirty-six groups in 30 studies reported about the duration of hospital stay following the UAE. The overall mean time of hospitalization was 9.044 days; 95% CI [8.028, 10.060], and the analysis was heterogeneous (*p* < 0.001, I^2^ = 99%).

**Figure 2:**
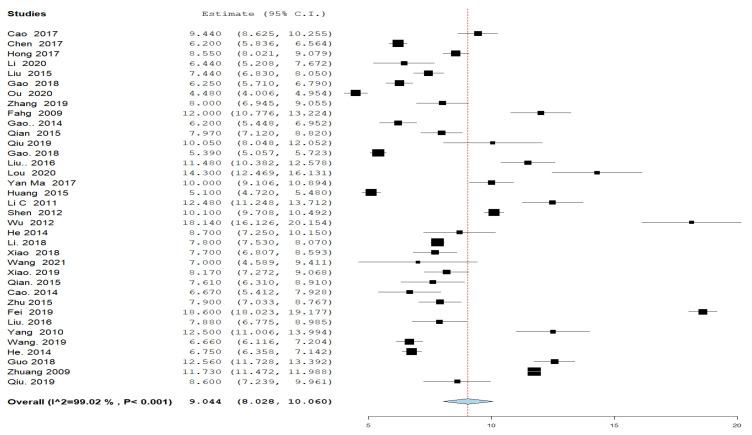
A forest plot of the duration of postprocedural hospital stay in days.

#### 3.4.3. Amount of Intraprocedural Blood Loss (Figure 3)

Twenty studies with 24 variable groups reported the amount of intraprocedural blood loss. The overall effect estimate of the intraprocedural amount of bleeding was 41.881 mL; 95% CI [34.102, 49.661], and the analysis was heterogeneous (*p* < 0.001, I^2^ = 99%).

**Figure 3:**
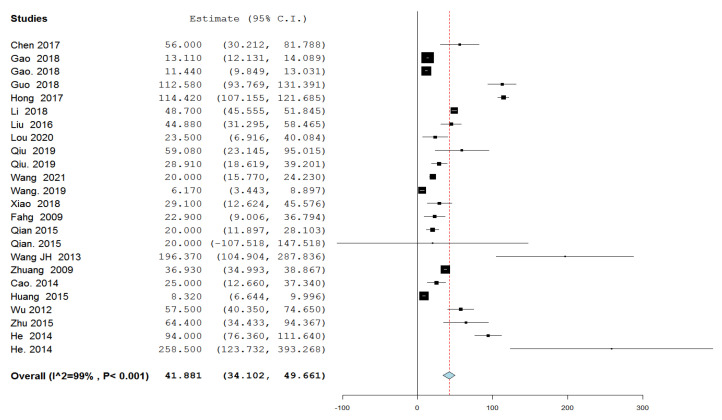
A forest plot of the amount of intraprocedural blood loss in mL.

#### 3.4.4. Success Rate (Figure 4)

All included studies reported success rate. The overall UAE success rate was 0.934; 95% CI [0.918, 0.951], and the analysis was heterogeneous (*p* < 0.001, I^2^ = 84%).

**Figure 4:**
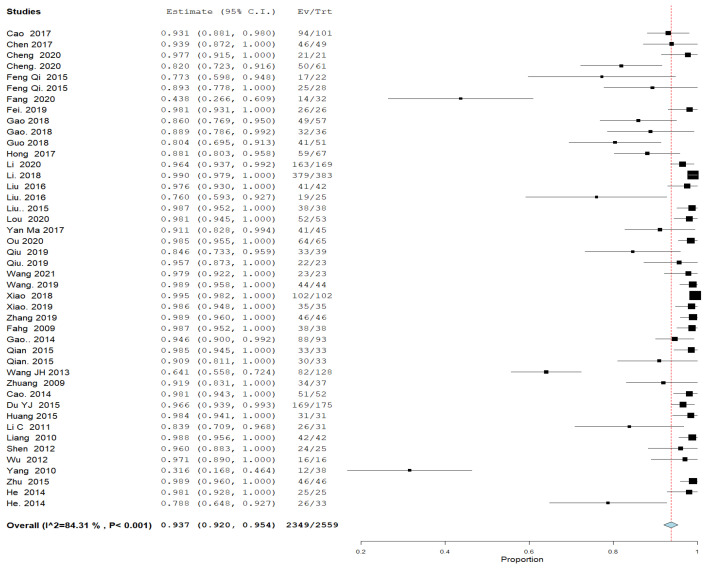
A forest plot of UAE success rate.

#### 3.4.5. Severe Complication Rate (Figure 5)

All included studies reported a severe complication rate. The overall proportion of severe complication rate was 0.012; 95% CI [0.008, 0.017], and the analysis was homogenous (*p* = 0.127, I^2^ = 19%).

**Figure 5:**
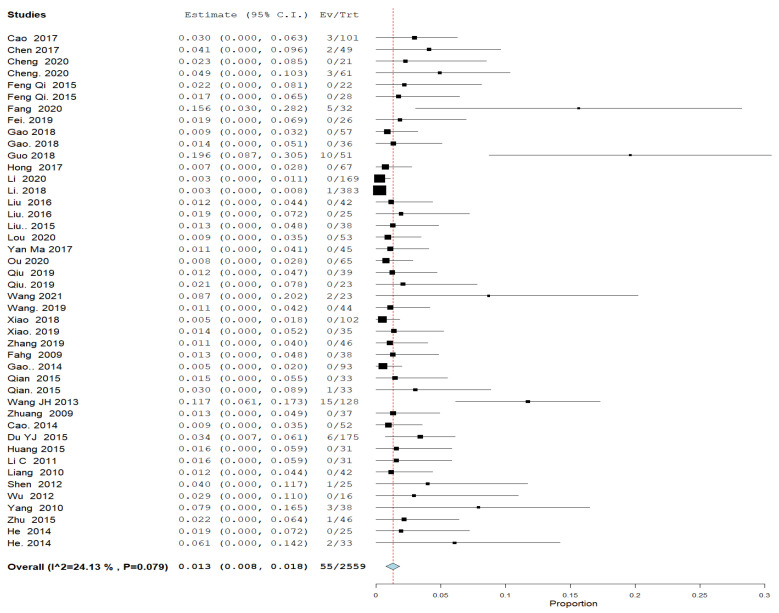
A forest plot of the severe complication rate.

## 4. Discussion

We analyzed data of 2655 CSP patients treated with UAE as part of first-line management. The results show that UAE was associated with a mean of 30.3 days for β-hCG normalization, a mean hospitalization time of 8.9 days, a mean intraprocedural blood loss of 41.9 mL, a success rate of 93.4%, and a severe complication rate of 1.2%.

Regarding the time for serum β-hCG normalization, it was found to be about 30.3 days. Qiao et al. [55] reported in their meta-analysis comparing adjuvant therapies to D&C, that UAE plus D&C had a shorter β-hCG normalization period than MTX plus D&C, which supports our results and shows that UAE may help decrease the β-hCG normalization time. Other studies have also shown a shorter normalization time after UAE compared with other treatments [28,30,36,51].

The mean amount of blood loss was 41.9 mL in our study. A recent systematic review and meta-analysis of MTX therapy for CSP reported that the mean blood loss was 76.3 mL [56], which strongly indicates that UAE probably helps decrease bleeding. Other studies also reported that the addition of UAE to the treatment protocol led to less bleeding [11,14,54,57,58].

In this study, UAE was associated with a mean post procedural hospital stay of 8.9 days. A recent meta-analysis of MTX for CSP showed an average stay of 11.7 days when MTX was used on an inpatient basis as a solo agent for CSP [56]. Another study found that UAE followed by D&C had significantly less hospital stay than MTX plus D&C [55].

The success rate of UAE in our study was 93.4%, which means that about 2432 patients managed with UAE as a part of their therapy needed no additional follow-up treatments. The success rate for MTX combined treatment was lower and equaling 90.7% [56].

Fifty-five patients (1.2%) managed by UAE reported events of severe complications. Forty-three of them suffered bleeding more than 500 mL or received a blood transfusion, fifteen underwent a hysterectomy, and fourteen had laparotomy. Petersen et al. reported a severe complication rate in different modalities, reaching 3.4% in UAE plus D&C, 1.2% in UAE plus D&C and hysteroscopy, and 2.8% in UAE plus D&C and MTX, and higher proportions for other modalities devoid of UAE [59].

### 4.1. Limitations

Despite being the first study to group all available research on UAE in China as part of CSP treatment, our study has many limitations. The wide variety of treatment options used in the included studies makes summarizing the results challenging and prohibits any meaningful combination of protocols to show that any one regiment is superior or even notably efficacious. Therefore, our recommendations are somewhat limited as we are combining many different protocols, all of which included UAE as part of the primary treatment of CSP. The small sample size for some studies and heterogeneity in the results are also major limitations. Most studies were of fair rating of quality, so future research is needed with more structured study designs and a larger scale of participants to ensure the effectiveness of UAE in CSP treatment. Moreover, as our analysis looked at this novel technique’s usage only in China, some of our findings may not be applicable to other countries. Notable is the fact that many consider China’s outpatient care system to still be developing, which likely accounts for the very long inpatient stays associated with these studies. This would not be expected in most developed countries with robust outpatient care systems, and further limits the versatility of this data.

### 4.2. Strengths

This was the first meta-analysis to look at all the available research on the use of UAE for CSP coming from China, and we were able to find a very wide breadth of studies to include. Therefore, although the usefulness of this data in the rest of the world may be limited, we provide researchers and physicians outside of China a first look at a novel practice that is likely very foreign to their own modes of practice. In addition, we used strict adherence to PRISMA guidelines, and were able to solve heterogeneity in most situations. Many gynecologists around the world may not previously have been aware that these novel techniques were being used in China.

## 5. Conclusions

China is producing a large amount of literature on the novel usage of UAE in the treatment of CSP. Although our study was limited by including many variations in protocols and modalities that were included with UAE in the treatment of CSP, we were able to calculate an overall success rate of approximately 93.4%, and a severe complication rate of approximately 1.2%. Because of conditions unique to the healthcare system in China, this data may have limited utility in application to patient care in other countries. More high-quality trials will be needed to further elucidate which exact treatment combinations and protocols yield the safest and most efficacious results for patients.

## Figures and Tables

**Table 1:** Shows the summary and baseline characteristics of the included studies.

ID	Year of Publication	Study Design	Inclusion Period	Primary Treatment Modality	Number of Cases	Age	Gestational Age (Days)	Success Rate (%)	Treatment Failure Causes	Severe Complications Rate (%)	Conclusion	Methodological Quality	Country of Origin
Cao 2017 [20]	2017	Retrospective cohort Study	2012–2016	UAE + curettage	101	32.98 (4.96)	–	93.07%	Treatment failure (n = 7) [underwent curettage again]	2.97%	Reduced menstrual blood volume can occur in scar pregnancy patients who received uterine artery embolization combined with curettage.	Fair	China
Chen 2017 [22]	2017	Retrospective cohort Study	2014–2016	UAE + curettage	49	33.7 (4.8)	–	93.90%	Treatment failure (n = 3) [underwent transvaginal hysterotomy]	4.08%	UAE combined with uterine curettage is less advantageous than transvaginal hysterotomy.	Fair	China
Cheng 2020 [23]	2020	Retrospective cohort Study	2010–2015	UAE + hysteroscopy	21	33.9 (1)	49 (45.5–65.5)	100%	–	0%	Compared with D&C ± UAE, LAOH ± UAE showed a higher success rate for CSP–II patients.	Fair	China
				UAE + D&C	61	33.5 (0.6)	52 (42–58)	82%	Treatment failure (n = 3) [laparoscopic surgery or laparotomy]	4.90%			
Qi 2015 [41]	2015	Case series	2009–2013	UAE + MTX + D&C	22	31.68 (4.58)	59.86 (17.67)	77.30%	Treatment failure (n = 8) additional intra–amniotic MTX injection or systemic MTX + D&C (n = 2), hysterotomy (n = 1). severe vaginal bleeding during curettage (n = 4) [hysterotomy] gelatin sponge separated and embolized the right leg (n = 1) [a second UAE.]	0%	UAE with or without local MTX infusion might be an effective treatment for CSP.	Good	China
				UAE + curettage	28	31.68 (4.58)	54.33 (17.51)	89.30%		0%			
Fang 2020 [26]	2020	Case series	2010–2016	UAE + curettage	32	–	68.05 (23.29)	43.75%	Treatment failure (n = 18) Massive vaginal bleeding (n = 5) [received blood transfusions and laparoscopy or laparotomy] large gestational sac (n = 13) [underwent surgery]	27.78%	CSP patients with short gestational age and small gestational sac can be treated with surgery, UAE, and HIFU and achieve safe and effective therapeutic effects.	Good	China
Fei 2019 [27]	2019	Retrospective cohort Study	2008–2017	UAE + MTX	26	31.4 (4.4)	–	100%	–	0%	There is no universal agreement on the optimal treatment modality for CSP.	Fair	China
Gao 2018 [14]	2018	Retrospective cohort study	2010–2015	UAE + curettage	57	33.46 (4.47)	54.25 (11.6)	86%	Treatment failure (n = 5) [underwent a repeat curettage or intrauterine packing with a water balloon]	0%	Adding intra–arterial MTX to UAE and curettage significantly promoted postoperative recovery, though success rate and bleeding events were not significantly affected.	Fair	China
				UAE + MTX +D&C	36	32.18 (5.65)	55.58 (9.82)	88.90%	Treatment failure (n = 2) [underwent a repeat curettage and intrauterine packing with a water balloon]	0%			
Guo 2018 [15]	2018	Retrospective cohort Study	2012–2017	UAE	51	32.21 (5.68)	54.82 (9.27)	80.40%	Treatment failure (n = 10) laparotomy hysterectomy (n = 5) LCSPDS operation (n = 3) scar lesion removal by abdominal incision (n = 2)	9.8% (5/51)	UAE and LCSPDS each have their advantages and disadvantages in treating CSP. Thus, appropriate individualized surgical programs based on specific patient circumstances are needed to avoid indiscriminately performing complete uterine cavity curettage.	Fair	China
Hong 2017 [30]	2017	Retrospective cohort Study	2014–2016	UAE + curettage	67	31.74 (3.69)	–	88.06%	_	0%	UAE combined with suction curettage under hysteroscopy is safe and effective in the management of CSP.	Fair	China
Li 2020 [32]	2020	Retrospective cohort Study	2013–2017	UAE + curettage	169	33.58 (4.88)	–	96%	Treatment failure (n = 6) repeated curettage (n = 2) resection of gestational tissues (n = 2) hemostatic drugs (n = 2)	0%	UAE combined with curettage treatment in CSP patients demonstrates a favorable success rate, which can also reduce MBV and proceeding pregnancy rate.	Fair	China
Li 2018 [33]	2018	Retrospective cohort Study	2006–2016	UACE + curettage + MTX	383	32.3 (4.9)	–	99%	Treatment failure (n = 4) massive blood loss of (n = 1) [systemic methotrexate] residual tissues (n = 3)[underwent hysteroscopic or transabdominal resection]	0.26%	UACE combined with curettage was found to be an effective fertility–sparing treatment for CSP. Further, the approach did not seem to harm future reproductive ability.	Poor	China
Liu 2016 [36]	2016	Retrospective cohort Study	2014–2016	UAE + MTX + D&C	42	32.43 (4.2)	–	97.50%	Treatment failure (n = 1) [needed additional treatment.]	0.00%	The combination of UAE, local MTX injection, and D&C for CSP patients is the optimal treatment strategy.	Fair	China
				UAE + MTX	25	32.44 (6.16)	–	76%	Treatment failure (n = 6) [required additional systemic MTX or D&C]	0%			
Liu 2015 [35]	2015	Retrospective cohort Study	2005–2013	UAE + curettage	38	33.42 (5.29)	55.42 (14.28)	100%	–	0%	UAE combined with curettage appears to be superior to MTX plus curettage for treatment of CSP with high serum b–hCG level.	Fair	China
Lou 2020 [37]	2020	Retrospective cohort Study	2013–2015	UAE + MTX + D&C	53	33 (3.6)	47 (8.4)	97.90%	Treatment failure (n = 1) [emergency UAE + Curettage]	0%	Pretreatment with MTX and UAE prior to curettage is safe and effective for the management of CSP.	Fair	China
Ma 2017 [38]	2017	Retrospective cohort Study	2012–2016	UAE + MTX + D&C	45	33 (6)	–	91.10%	Treatment failure (n = 4) systemic and local MTX therapy + curettage (n = 1) [supplementary MTX therapy] (n = 2) abdominal CSP mass resection (n = 1)	0%	All treatments have high success rates and no significant effects on intraoperative bleeding.	Fair	China
Ou 2020 [39]	2020	Prospective cohort study	2016–2018	UAE + curettage	65	34 (4.4)	52.29 (10.32)	98.46%	Treatment failure (n = 1) [repeat curettage]	0%	Suction and curettage alone is a more suitable option than UAE followed by suction and curettage.	Fair	China
Qiu 2019 [43]	2019	Retrospective cohort Study	2013–2018	UAE + curettage	39	32.1 (5.02)	_	84.60%	Treatment failure (n = 6) Massive vaginal bleeding (n = 3) [hysteroscopy or iodoform gauze packing.] unsatisfactory decrease in serum β–HCG level (n = 3) [received intramuscular injection of MTX] retained products of conception (n = 3) [underwent hysteroscopy]	0%	D&C guided by ultrasonography after UAE treatment showed good clinical efficacy.	Fair	China
				UAE + hysteroscopy	23	32.48 (4.73)	_	95.70%	Treatment failure (n = 1) Massive vaginal bleeding received [iodoform gauze packing]	0%	Hysteroscopy after UAE treatment showed good clinical efficacy.		
Wang 2021 [16]	2021	Retrospective cohort study	2017–2019	UAE+ D&C + Hysteroscopy	23	29.2 (3.6)	_	100%	_	8.70%	UAE pretreatment method seems to be effective, economical, and with few side effects in the management of CSP.	Fair	China
Wang 2019 [50]	2019	Retrospective cohort study	2016–2018	UAE + MTX + hysteroscopy	44	31.84 (2.47)	_	100%	_	0%	UAE can effectively reduce intraoperative blood loss but increases the risk of postoperative complications, length of hospital stay, medical costs.	Fair	China
Xiao 2018 [48]	2018	Retrospective cohort study	2011–2014	UACE + curettage + MTX	102	33.1 (4.6)	51.19 (11.13)	100%	_	0%	UACE combined with D&C is a useful measure for most Type 2 CSP cases in the first trimester. For Type 2 CSP cases in the second trimester, UACE before laparotomy could be a reasonable choice.	Fair	China
Xiao 2019 [49]	2019	Retrospective case–control study	2014–2017	UAE + D&C + hysteroscopy	35	32.67 (6.96)	52.5 (13.91)	100%	_	0%	combination of UAE and surgery should be selected carefully because of its potential fertility complication.	Fair	China
Zhang 2019 [52]	2019	Retrospective cohort study	_	UAE + curettage	46	32.5 (4.7)	48.7 (9.8)	100%	_	0%	Compared to UAE, lauromacrogol–based sclerotherapy is a safe, effective, and economic approach in the pretreatment for uterine scar pregnancy.	Fair	China
Fahg 2009 [25]	2009	Prospective cohort study	2004–2088	UAE + curettage	38	32.5 (4.8)	53.35 (7.72)	100%	_	0%	UAE followed by curettage is recommended to medical facilities where UAE is available.	Fair	China
Gao 2014 [28]	2014	Prospective cohort study	2009–2012	UAE + curettage	93	33.4 (4.5)	49.84 (7.72)	94.62%	Treatment failure (n = 5) [needed additional interventions]	0%	UAE combined with D&C within 24 hours was an effective and safe uterine preservation treatment for CSP.	Fair	China
Qian 2015 [42]	2015	RCT	2008–2013	UAE + curettage	33	30.79 (4.29)	51.33 (7.57)	100%	_	0%	UAE plus curettage was successful in terminating a gestational sac type of CSP.	High	China
				UAE + D&C + hysteroscopy	33	32 (4.15)	52 (11.14)	90.91%	Treatment failure (n = 3) hemorrhage during surgery (n = 1) [Emergency hysterectomy] additional MTX therapy (n = 2)	3.03%			
Wang 2013 [47]	2013	Retrospective cohort study	2007–2012	UAE + curettage	128	32.28 (4.76)	48.64 (7.98)	88.28%	Treatment failure (n = 15) Emergency hysterectomy (n = 5)	11.72%	For CSP masses with a GA of 8 weeks or more and a diameter of 6 cm or more, the outcome of surgical evacuation after UAE tends to be unsatisfactory.	Fair	China
Zhuang 2009 [54]	2009	RCT	2003–2007	UAE + curettage	37	32.23 (.65)	51.24 (1.4)	91.89%	Treatment failure (n = 3) Iodoform meche (n = 1) Readmitted due to bleeding (n = 2)	0%	UAE followed by suction curettage seems to have more advantages than systemic MTX treatment and maybe a priority option.	Moderate	China
Cao 2014 [21]	2014	Retrospective cohort study	2007–2012	UAE + D&C + hysteroscopy	52	33.3 (4.5)	49.13 (14.74)	98.08%	Treatment failure (n = 1) [Resection of the lower uterine segment]	0%	UAE combined with D&C is a safe and efficient treatment for CSP.	Fair	China
Du 2015 [24]	2015	Retrospective cohort study	2006–2012	UAE + MTX + D&C	175	32.44 (4.6)	54.05 (14.04)	96.57%	Treatment failure (n = 6) tamponade with iodoform gauze packs or an inflated balloon catheter (n = 3) emergency local CSP resection via laparotomy (n = 1) Emergency hysterectomy (n = 2)	3.43%	Increased gestational age increases the risk of bleeding in CSP treated by UAE+MTX+D&C.	Fair	China
Huang 2015 [31]	2015	Retrospective cohort study	2009–2014	UAE + MTX + D&C	31	32.42 (5.94)	42.12 (6.32)	100%	_	0%	UAE combined with MTX is a safe and efficient treatment of CSP.	Fair	China
Li 2011 [11]	2011	RCT	2002–2009	UAE + MTX + D&C	31	34.15 (5.41)	70.89 (35.94)	83.87%	Treatment failure (n = 5) tamponade with iodoform gauze (n = 2) re–embolization (n = 3)	0%	Arterial chemoembolization with MTX was more effective than systemic MTX treatment for termination of CSP.	Low	China
Liang 2010 [34]	2010	Retrospective cohort study	2005–2009	UAE + MTX + D&C	42	31.3 (3.6)	5–10.5 weeks	100%	_	0%	The use of UAE for the treatment of CSP is tolerated well and has few complications.	Poor	China
Shen 2012 [44]	2012	Retrospective cohort study	2008–2010	UAE + MTX + D&C	25	32.7 (6)	55.45 (2.11)	96.00%	Treatment failure (n = 1) [Hysterectomy]	4.00%	UAE and MTX appears to be a safe and effective treatment for CSP and causes less morbidity than current approaches.	Fair	China
Wu 2012 [13]	2012	Retrospective cohort study	2000–2010	UAE + MTX + D&C	16	33.09 (4.33)	48.18 (11.68)	100%	_	0%	UAE combined with intraarterial MTX infusion could be an effective and safe treatment for CSP.	Fair	China
Yang 2010 [51]	2010	Retrospective cohort study	2003–2008	UAE + MTX	38	31.5 (7.25)	47.73 (11.1)	31.58%	Treatment failure (n = 26) Re–embolization (n = 2) Additional D&C (n = 24)	7.89%	UAE combined with local MTX benefits women wishing to preserve fertility and is suitable for use as the primary treatment for CSP.	Fair	China
Zhu 2015 [53]	2015	Retrospective cohort study	2014	UAE + D&C + hysteroscopy	46	31.4 (5.1)	60.6 (16.4)	100%	_	2.17%	UAE combined with suction curettage under hysteroscopic guidance is safe and effective in treating patients with CSP.	Fair	China
He 2014 [29]	2014	Retrospective cohort study	2005–2010	UAE + MTX + hysteroscopy	25	_	_	100%	_	0%	Combination of laparoscopy and hysteroscopy is much safer and more effective than uterine curettage as a supplementary measure following the UAE management of CSP.	Fair	China
				UAE + MTX + D&C	33			78.79%	Treatment failure (n = 7) [underwent multiple curettages]	6.10%			

SD, standard deviation; MTX, methotrexate; D & C, dilatation and curettage; UAE, uterine artery embolization; CSP, cesarean scar pregnancy; LAOH, laparoscopy–assisted by operative hysteroscopy; LCSPDS, laparoscopic cesarean scar pregnancy debridement surgery; HIFU, high–intensity focused ultrasound; SCEM, selective chemoembolization with methotrexate; GA, gestational age; MBV, menstrual blood volume; UAC, uterine artery chemoembolization.

## Data Availability

All supporting data is included or referenced in this manuscript. The authors have no additional data used in this study.

## Supplementary Materials

The following supporting information can be downloaded at: https://www.mdpi.com/article/10.3390/jcm11247393/s1, Figure S1: PRISMA flow chart summarizing the process of study selection; Table S1: The Risk of Bias for all included Cohort studies (by NIH tool); Table S2: Risk of Bias for all included Case series studies (by NIH tool).

## Commitment to Diversity

The Marchand Institute remains committed to diversity and tolerance in its research, and actively maintains a workplace free of racism and sexism. Greater than half of the authors for this study are female and many represent diverse backgrounds and under-represented ethnic groups.
