# Supplementary material for: Treatment of Cesarean Scar Ectopic Pregnancy in China with Uterine Artery Embolization—A Systematic Review and Meta-Analysis
Source: J Clin Med. 2022 Dec 13;11(24):7393. doi: 10.3390/jcm11247393 (PMC9783593; doi:10.3390/jcm11247393)
Supplement: Supplementary file 1 [file jcm-11-07393-s001.zip › jcm-2015758-supplementary.pdf]

Supplementary Materials

Treatment of Cesarean Scar Ectopic Pregnancy in China with Uterine Artery Embolization—A Systematic Review and Meta-Analysis

Greg J. Marchand [1,*], Ahmed Taher Masoud [1,2], Catherine Coriell [1], Hollie Ulibarri [1], Julia Parise [1], Amanda Arroyo [1], Sydnee Goetz [1], Carmen Moir [1], Atley Moberly [1] and Malini Govindan [1]

[1] Marchand Institute for Minimally Invasive Surgery, Mesa, AZ 85209, USA

[2] Faculty of Medicine, Fayoum University, Fayoum 63514, Egypt

* Correspondence: gm@marchandinstitute.org

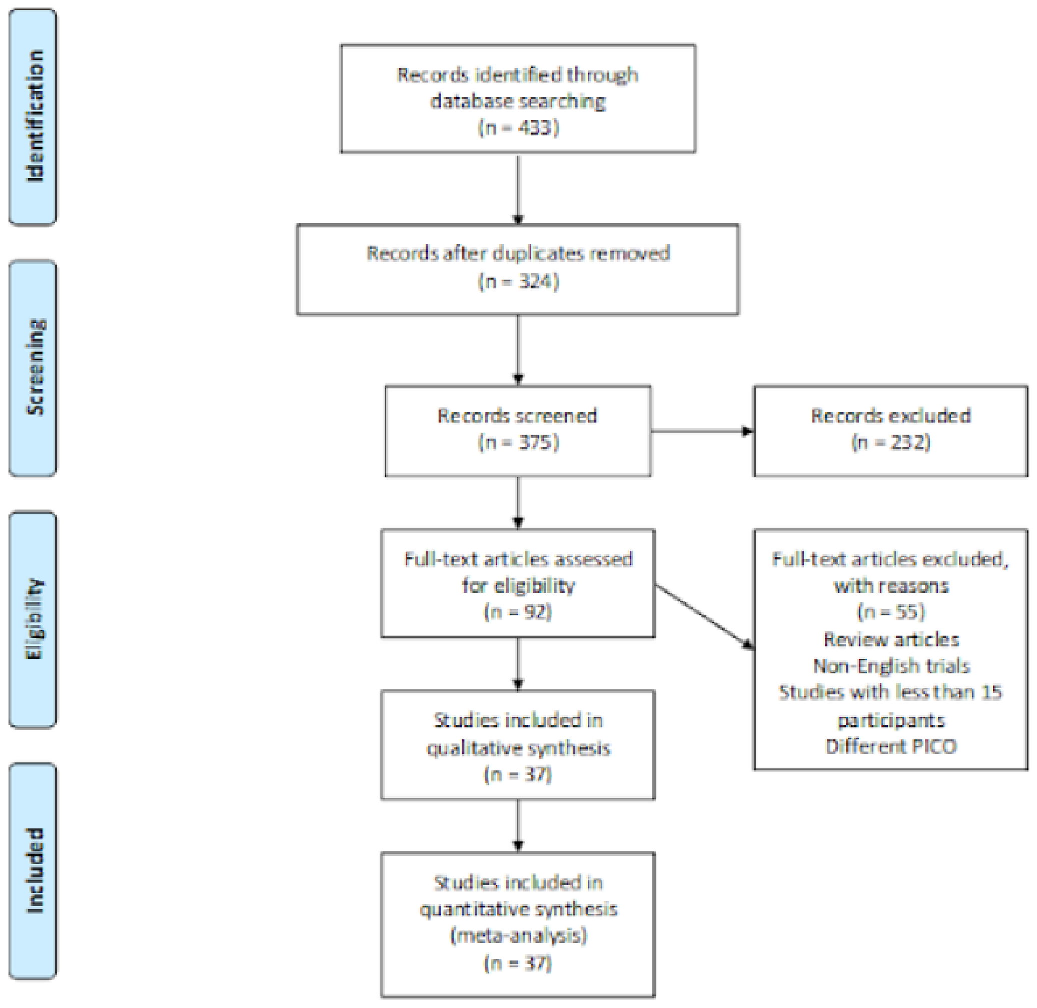

Supplementary Figure S1. PRISMA flow chart summarizing the process of study selection.

Supplemental Table S1

Supplemental Table S1: The Risk of Bias for all included Cohort studies (by NIH tool)

ID	1	2	3	4	5	6	7	8	9	10	11	12	13	14	Total score	Quality rating
Cao 2017 [20]	Yes	Yes	NA	No	No	yes	yes	NA	Yes	NA	Yes	NA	Yes	no	8.5	Fair
Chen 2017 [22]	Yes	Yes	NA	No	No	yes	yes	NA	Yes	NA	Yes	NA	Yes	no	8.5	Fair
Cheng 2020 [23]	Yes	Yes	NA	No	No	yes	yes	NA	Yes	NA	Yes	NA	Yes	no	8.5	Fair
Fei 2019 [27]	Yes	Yes	NA	No	No	yes	yes	NA	Yes	NA	Yes	NA	Yes	Yes	9	Fair
Gao 2018 [14]	Yes	Yes	NA	Yes	yes	yes	yes	NA	Yes	NA	Yes	NA	Yes	Yes	10	Fair
Guo 2018 [18]	Yes	Yes	NA	Yes	No	yes	yes	NA	Yes	NA	Yes	NA	Yes	Yes	9.5	Fair
Hong 2017 [30]	Yes	Yes	NA	No	No	yes	yes	NA	Yes	NA	Yes	NA	Yes	Yes	9	Fair
Li 2020 [32]	Yes	Yes	NA	Yes	No	yes	yes	NA	Yes	NA	Yes	NA	Yes	Yes	9.5	Fair
Li 2018 [33]	Yes	No	NA	No	No	yes	yes	NA	Yes	NA	Yes	NA	No	Yes	8	Poor
Liu 2016 [36]	Yes	No	Yes	No	No	yes	yes	NA	Yes	NA	Yes	NA	Yes	no	9	Fair
Liu 2015 [35]	Yes	Yes	NA	No	No	yes	yes	NA	Yes	NA	Yes	NA	Yes	no	8.5	Fair
Lou 2020 [37]	Yes	Yes	NA	No	No	yes	yes	NA	Yes	NA	Yes	NA	Yes	no	8.5	Fair
Ma 2017 [38]	Yes	Yes	NA	No	No	yes	yes	NA	Yes	NA	Yes	NA	Yes	Yes	9	Fair
Ou 2020 [39]	Yes	Yes	Yes	No	No	yes	yes	NA	Yes	NA	Yes	NA	Yes	Yes	10	Fair
Pyra 2021 [40]	Yes	No	NA	No	No	yes	yes	NA	Yes	NA	Yes	NA	Yes	no	8	Poor
Qiu 2019 [43]	Yes	Yes	Yes	Yes	No	yes	yes	NA	Yes	NA	Yes	NA	Yes	no	10	Fair
Wang 2021 [16]	Yes	Yes	NA	No	No	yes	yes	NA	Yes	NA	Yes	NA	Yes	no	8.5	Fair
Wang 2019 [50]	Yes	Yes	NA	No	No	yes	yes	NA	Yes	NA	Yes	NA	Yes	no	8.5	Fair
Xiao 2018 [48]	Yes	Yes	NA	No	No	yes	yes	NA	Yes	NA	Yes	NA	Yes	no	8.5	Fair
Xiao 2019 [49]	Yes	Yes	NA	Yes	No	yes	yes	NA	Yes	NA	Yes	NA	Yes	no	9	Fair
Zhang 2019 [52]	Yes	Yes	NA	No	No	yes	yes	NA	Yes	NA	Yes	NA	Yes	no	8.5	Fair
Fahg 2009 [25]	Yes	Yes	NA	No	No	yes	yes	NA	Yes	NA	Yes	NA	Yes	no	8.5	Fair
Gao 2014 [28]	Yes	Yes	Yes	Yes	No	yes	yes	NA	Yes	NA	Yes	NA	Yes	no	10	Fair
Wang 2013 [47]	Yes	Yes	NA	No	No	yes	yes	NA	Yes	NA	Yes	NA	Yes	Yes	9	Fair
Cao 2014 [21]	Yes	Yes	NA	No	No	yes	yes	NA	Yes	NA	Yes	NA	Yes	no	8.5	Fair
Du 2015 [24]	Yes	Yes	Yes	Yes	No	yes	yes	NA	Yes	NA	Yes	NA	Yes	Yes	10.5	Fair
Huang 2015 [31]	Yes	Yes	NA	No	No	yes	yes	NA	Yes	NA	Yes	NA	Yes	no	8.5	Fair
Liang 2010 [34]	Yes	Yes	NA	No	No	yes	yes	NA	Yes	NA	No	NA	Yes	no	8	Poor
Shen 2012 [44]	Yes	Yes	NA	No	No	yes	yes	NA	Yes	NA	Yes	NA	Yes	no	8.5	Fair
Wu 2012 [13]	Yes	Yes	NA	Yes	No	yes	yes	NA	Yes	NA	Yes	NA	Yes	no	9	Fair
Yang 2010 [51]	Yes	Yes	NA	No	No	yes	yes	NA	Yes	NA	Yes	NA	Yes	Yes	9	Fair
Zhu 2015 [53]	Yes	Yes	NA	Yes	No	yes	yes	NA	Yes	NA	Yes	NA	Yes	no	9	Fair
He 2014 [29]	Yes	Yes	NA	Yes	No	yes	yes	NA	Yes	NA	Yes	NA	Yes	no	9	Fair
Wang 2021 [16]	Yes	Yes	NA	No	No	yes	yes	NA	Yes	NA	Yes	NA	Yes	no	8.5	Fair
Wang 2019 [50]	Yes	Yes	NA	No	No	yes	yes	NA	Yes	NA	Yes	NA	Yes	no	8.5	Fair

NA: Not applicable

1: Was the research question or objective in this paper clearly stated?

2: Was the study population clearly specified and defined?

3: Was the participation rate of eligible persons at least 50%?

4: Were all the subjects selected or recruited from the same or similar populations (including the same time period)? Were inclusion and exclusion criteria for being in the study prespecified and applied uniformly to all participants?

5: Was a sample size justification, power description, or variance and effect estimates provided?

6: For the analyses in this paper, were the exposure(s) of interest measured prior to the outcome(s) being measured?

7: Was the time frame sufficient so that one could reasonably expect to see an association between exposure and outcome if it existed?

8: For exposures that can vary in amount or level, did the study examine different levels of the exposure as related to the outcome (eg, categories of exposure, or exposure measured as continuous variable)?

9: Were the exposure measures (independent variables) clearly defined, valid, reliable, and implemented consistently across all study participants?

10: Was the exposure(s) assessed more than once over time?

11: Were the outcome measures (dependent variables) clearly defined, valid, reliable, and implemented consistently across all study participants?

12: Were the outcome assessors blinded to the exposure status of participants?

13: Was there loss to follow-up after baseline 20% or less?

14: Were key potential confounding variables measured and adjusted statistically for their impact on the relationship between exposure(s) and outcome(s)?

Supplemental Table S2

Supplemental Table S2: Risk of Bias for all included Case series studies (by NIH tool)

ID	1	2	3	4	5	6	7	8	9	Total scores	Quality rating
Qi 2015 [41]	Yes	Yes	Yes	Yes	Yes	Yes	Yes	Yes	Yes	9	Good
Fang 2020 [26]	Yes	Yes	Yes	Yes	Yes	Yes	Yes	Yes	Yes	9	Good

1: Was the study question or objective clearly stated?

2: Was the study population clearly and fully described, including a case definition?

3: Were the cases consecutive?

4: Were the subjects comparable?

5: Was the intervention clearly described?

6: Were the outcome measures clearly defined, valid, reliable, and implemented consistently across all study participants?

7: Was the length of follow-up adequate?

8: Were the statistical methods well-described?

9: Were the results well-described?
